# *Candida utilis* Ameliorates Dextran Sulfate Sodium-Induced Colitis in Mice via NF-κB/MAPK Suppression and Gut Microbiota Modulation

**DOI:** 10.3390/ijms26051993

**Published:** 2025-02-25

**Authors:** Rongxin Zang, Zhouliang Liu, Huihao Wu, Wenyan Chen, Rui Zhou, Fazheng Yu, Yaodong Li, Hongwei Xu

**Affiliations:** 1 College of Life Science and Engineering, Northwest Minzu University, Lanzhou 730100, China; 2 Engineering Research Center of Key Technology and Industrialization of Cell-Based Vaccine, Ministry of Education, Lanzhou 730030, China; 3 Key Laboratory of Biotechnology and Bioengineering of State Ethnic Affairs Commission, Northwest Minzu University, Lanzhou 730030, China; wuhuihao99@163.com; 4 State Key Laboratory for Diagnosis and Treatment of Severe Zoonotic Infectious Diseases, Key Laboratory for Zoonosis Research of the Ministry of Education, Institute of Zoonosis, College of Veterinary Medicine, Jilin University, Changchun 130062, China; 5 Gansu Tech Innovation Center of Animal Cell, Northwest Minzu University, Lanzhou 730030, China

**Keywords:** ulcerative colitis, tight junction proteins, short-chain fatty acids (SCFAs), nuclear factor-kappa B (NF-ĸB)

## Abstract

*Candida utilis* (CUM) possesses various biological effects, including anti-inflammatory, intestinal microbiota regulatory, and immunomodulatory activities. However, there has been little exploration regarding the effects of CUM on ulcerative colitis (UC). Therefore, this study aimed to investigate the beneficial effects of CUM on alleviating dextran sulfate sodium (DSS)-induced UC in mice and to explore the potential underlying mechanisms. Here, the effect of CUM on UC was analyzed using a DSS-induced colitis mouse model (n = 9), the results of which indicated a decrease in disease activity index (DAI) in DSS-induced UC mice. Furthermore, CUM alleviated colon shortening, minimized intestinal tissue damage, and preserved intestinal tight junction proteins (Claudin-3, Occludin, and ZO-1). CUM reduced the level of pro-inflammatory cytokines (IL-1β, IL-6, and TNF-α), inhibited the activation of the NF-ĸB, MAPK and PPARγ signaling pathways, and decreased the level of oxidative mediators (MPO, SOD and MDA) in the colon of UC mice. Additionally, it mitigated the dysbiosis of intestinal microbiota in UC mice by increasing the abundance of *Prevotellaceae* and *Lactobacillus* while decreasing the abundance of *Bacteroidaceae* and *Enterobacteriaceae*. CUM alleviated the decrease in short-chain fatty acids (SCFAs) content in the colon of UC mice. The above results provide a scientific basis for CUM, as a natural supplement, to restore the balance of the gut inflammatory microbiota and promote gut health.

## 1. Introduction

Ulcerative colitis (UC) is a chronic, nonspecific inflammatory bowel disease of unknown etiology that is characterized by persistent inflammation of the colonic mucosa [1]. Its clinical symptoms mainly include diarrhea, abdominal pain, and mucopurulent bloody stools [2]. The incidence of UC is on the rise globally. UC not only causes patients persistent and painful symptoms such as abdominal pain, diarrhea, and bloody stools, but may also trigger a series of serious complications, like colon cancer, which poses a great threat to patients’ lives and health. This makes the research on UC urgent and important, attracting extensive medical attention. Currently, some immunosuppressants and anti-inflammatory drugs, such as corticosteroids, 5-aminosalicylic acid, and sulfasalazine, are the main drugs for clinical control of UC symptoms [3]. However, long-term use of these drugs can lead to serious side effects, greatly limiting their clinical application [4]. Therefore, there is still an urgent need to develop a new drug that is highly effective, has minimal side effects, and targets multiple pathways for the treatment of UC.

The gastrointestinal tract is the largest immune organ in mammals, with main functions including digestion, absorption, and secretion of hormones and enzymes to regulate food intake and animal metabolism [5,6]. Changes in intestinal immune function can lead to a series of gastrointestinal diseases. The gut is home to trillions of microorganisms known as the microbiota. In addition to boosting immunity and alleviating metabolic syndrome, these microorganisms also play a role in metabolism and nutrient intake [7]. The intestinal mucosa covers the inner wall of the intestine, which not only aids in nutrient absorption but also acts as a barrier, preventing bacteria and harmful substances from entering the bloodstream [8]. The intestinal mucosa is also an important part of the immune system, helping the body fight pathogens. It serves as a communication hub between nutrient absorption and primary metabolites, as well as a strategy to enhance intestinal immunity against antigens and foreign substances [9,10].

Yeast have a promising application prospect in restoring intestinal integrity and mucosal barrier function through various pathways in the gastrointestinal tract [11,12]. Studies have demonstrated that using yeast as a carbon and energy source for the intestinal microbiota is essential for maintaining the integrity of the colonic mucus barrier [13]. Besides, yeast promote the growth of beneficial microorganisms in addition to their antioxidant, anti-proliferative and anti-inflammatory bioactivities [14]. CUM has been shown to promote the development of intestinal immune organs, humoral immunity, cellular immunity, and rapid removal of pathogens from the body, as well as improve the efficiency of the immune system and enhance the resistance of animals to various diseases [15,16]. However, given the limited research on the therapeutic effects of CUM on DSS-induced UC and mucosal barrier damage, this study aimed to analyze the effects of CUM on a dextran sulfate sodium salt (DSS)-induced mouse model of UC and to explore its potential therapeutic effects and mechanisms of action. While exploring the treatment and protective mechanisms of CUM on UC, we selected the NF-κB, MAPK, and PPARγ signaling pathways for further study. The NF-κB signaling pathway plays a crucial role in the regulation of the inflammatory response. Its over-activation can trigger the release of a series of pro-inflammatory factors, exacerbating the inflammatory state of UC. The MAPK signaling pathway is involved in cellular stress responses and inflammatory signal transduction, and is closely related to the onset and inflammatory progression of UC. The PPARγ signaling pathway is of great significance for maintaining intestinal barrier function and regulating the immune response, and its abnormal function is closely associated with the occurrence and development of UC. By studying the effects of CUM on these three signaling pathways, we revealed its protective mechanism against UC at the molecular level, providing a theoretical basis and new research directions for the development of more effective treatment strategies for UC.

## 2. Results

### 2.1. CUM Improved the Symptoms of DSS-Induced Colitis in Mice

**Figure 1:**
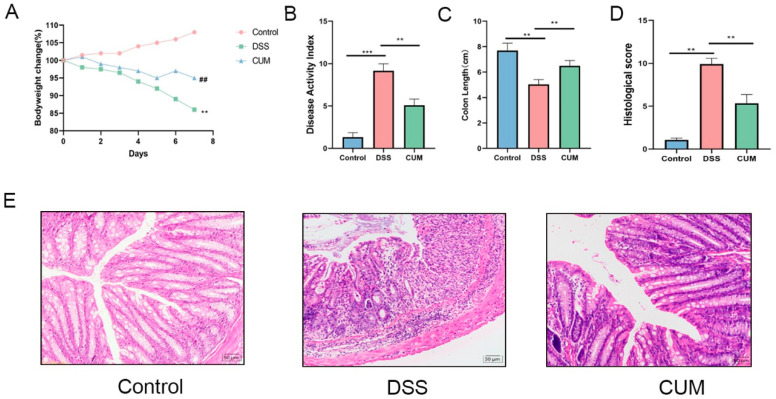
CUM treatment effects on DSS-induced colitis in mice. (**A**) Body weight change (%), (**B**) colon length, (**C**) disease activity index (DAI). (**D**) The colons from each experimental group were processed for histological evaluation. Representative histological changes of colons obtained from mice in different groups. (**E**) Histopathological scores. Data are presented as mean ± SD (n = 9). Significance = ** *p* < 0.01 Control vs. CUM group; ## *p* < 0.01 CUM vs. the DSS group; ** *p* < 0.01 and *** *p* < 0.001 indicate a significant difference compared with the DSS group.

In this experiment, the body weight and colon length were significantly decreased in the DSS group compared to the control group (Figure 1A,B) (*p* < 0.05). After administering CUM to UC mice, the disease state of the mice was improved significantly, as evidenced by the significant recovery in colon length and body weight and decrease in DAI (Figure 1C) (*p* < 0.05). In addition to changes in colon length, DSS also induced pathological damage to the colon tissue of the mice, as evidenced by the morphological images of colon tissue from the three experimental groups. As shown in Figure 1D, the colon morphology of healthy mice was intact, with a compact structure and no obvious damage. The colon morphology of the DSS group featured a sparse cell arrangement, mucosal tissue ulceration with mild bleeding, and significant infiltration of inflammatory cells into the mucosal layer. The administration of CUM improved the integrity of the colon tissue and reduced the number of inflammatory cells.

### 2.2. CUM Improved DSS-Induced Colonic Oxidative Stress

Compared with the control group, the levels of pro-inflammatory cytokines such as IL-1β, IL-6, IL-8 and TNF-α in the DSS group increased significantly (*p* < 0.05). After CUM treatment, the levels of pro-inflammatory cytokines in the colon tissues of mice were significantly lower than those in the DSS-treated group (*p* < 0.05). Therefore, CUM can significantly reduce the inflammatory cytokines in the colon tissues of DSS-induced colitis mice, indicating its good anti-inflammatory effect. 

**Figure 2:**
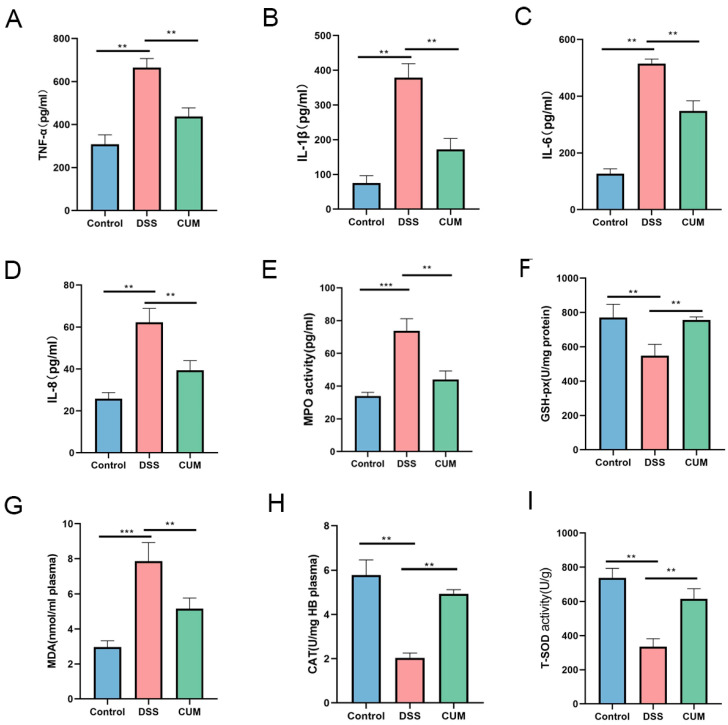
The impact of CUM on the expression of pro-inflammatory cytokines and oxidative mediators. (**A**) IL-1β, (**B**) IL-6, (**C**) TNF-α, (**D**) IL-8, (**E**) MPO, (**F**) GSH-PX, (**G**) MDA, (**H**) CAT, and (**I**) T-SOD. Data are presented as mean ± SD (n = 6). Significance = ** *p* < 0.01 and *** *p* < 0.001 indicate a significant difference compared with the DSS group.

Previous studies have revealed that many intestinal diseases are associated with reactive oxygen species or free radicals. CAT, SOD, and GSH-px are endogenous antioxidant enzymes in the body, which function to eliminate reactive oxygen species [6]. Therefore, the body’s ability to resist oxidative stress can be reflected by the activity of these enzymes. In this experiment, the activities of SOD, CAT, and GSH-px in the colon tissue of mice in the DSS group were significantly decreased compared to the control group. Consistent with previous reports, the content of MDA in the colon tissue of UC mice was significantly increased (*p* < 0.05). As shown in the figure, MPO increased following DSS induction (*p* < 0.05). The changes in the above indicators indicated that the mice developed oxidative damage and inflammation after DSS induction (Figure 2A–I). However, the intervention of CUM inhibited these changes, and these results suggested that CUM had a mitigating effect on colonic inflammation.

### 2.3. CUM Protects the Intestinal Mucosal Barrier in Mice

**Figure 3:**
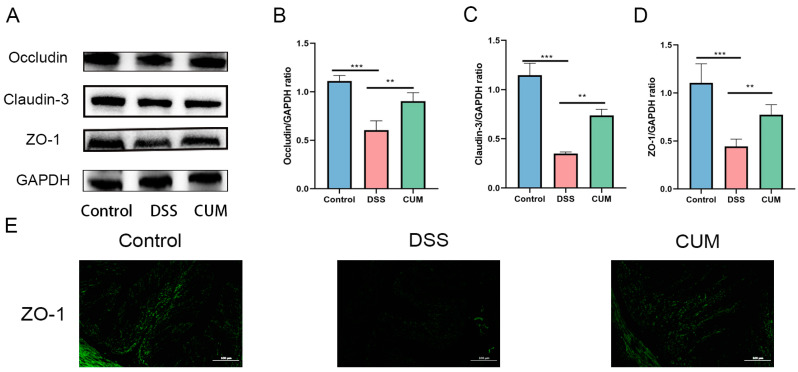
Effects of CUM supplementation on the level of tight junction proteins. (**A**) Protein expression of Occludin, Claudin-3, and ZO-1 in the three groups determined using Western blots. (**B**) The relative expression of Occludin was normalized to GAPDH; (**C**) the relative expression of Claudin-3 was normalized to GAPDH; (**D**) the relative expression of ZO-1 was normalized to GAPDH. (**E**) Representative images of Control, DSS, and CUM at the level of ZO-1 by immunofluorescence staining. The image was taken by a confocal microscope with ×100 magnification. Data are presented as mean ± SD (n = 6). Significance = ** *p* < 0.01 and *** *p* < 0.001 indicate a significant difference compared with the DSS group.

Tight junction (TJ) proteins are transmembrane proteins that are crucial for the function of the intestinal mucosal barrier. In patients with colitis, the intestines are inflamed, and the intestinal barrier function is weakened. Occludin, ZO-1 and Claudin-3 are core transmembrane proteins that constitute the TJs of the intestinal epithelium. By forming a selectively permeable barrier, they prevent the luminal-to-blood penetration of pathogens and toxins. Meanwhile, they regulate the paracellular transport of ions and small molecules. Therefore, regulating intestinal tight junction proteins and inflammation to restore the intestinal barrier function in patients may be a feasible therapeutic strategy. As shown in Figure 3A–E, DSS treatment significantly decreased the protein expression levels of ZO-1, Occludin, and Claudin-3 in the colon of mice in the DSS group (*p* < 0.05). Compared with the DSS group, the expression of ZO-1, Occludin, and Claudin-3 in mice of the CUM group increased significantly (*p* < 0.05). These results indicate that CUM can alleviate the intestinal barrier damage caused by DSS in mice.

### 2.4. The Impact of CUM on Colon Transcriptome and Key Pathways

**Figure 4:**
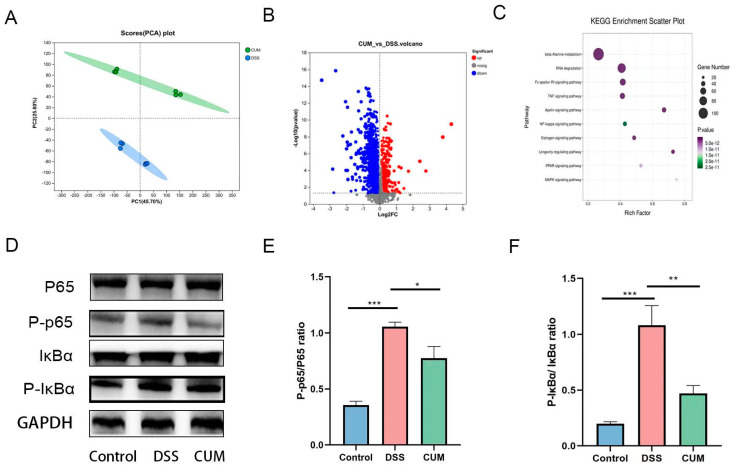
CUM protects against colitis via NF-κB inhibition. (**A**) Volcanic plot of metabolite changes; (**B**) Principal component analysis; (**C**) KEGG enrichment analysis; (**D**) Representative images show that CUM inhibits the activation of the NF-ĸB pathway. Quantitative results show that CUM inhibits the activation of NF-ĸB (**E**) and IκBα (**F**) in DSS-treated mice. Each value is expressed as mean ± SD (n = 6). Significance = * *p* < 0.05, ** *p* < 0.01 and *** *p* < 0.01 indicate a significant difference compared with the DSS group.

In order to investigate the mechanisms by which CUM affects colitis, we analyzed the colonic transcriptome profile (Figure 4A). According to the volcano plot of gene expression, compared to the DSS group, the CUM group showed 202 upregulated genes and 197 downregulated genes (Figure 4B). KEGG pathway analysis revealed involvement in cytokine–cytokine receptor interactions, the NFκB pathway, the MAPK signaling pathway, and the PPARγ pathway, among others (Figure 4C).

### 2.5. CUM Altered the Protein Expression in the Colon of UC Mice

According to prior research reports, PPARγ inhibits the activation of the MAPK and NF-ĸB signaling pathways, thereby regulating inflammation and apoptosis [17,18]. The PPARγ, MAPK, and NF-κB signaling pathways are closely associated with colitis. When activated, PPARγ inhibits the production of pro-inflammatory factors, regulates the intestinal barrier and microbiota, and exerts a protective effect. MAPKs are activated by inflammatory stimuli, promoting the expression of inflammatory genes, and participating in cell apoptosis and the regulation of immune cells, thus exacerbating inflammation. NF-κB initiates the transcription of inflammatory genes under inflammatory stimulation, regulates immune cells, affects the functions of intestinal epithelial cells, and contributes to the development of colitis. These three signaling pathways interact with each other and jointly regulate intestinal inflammation, immunity, and barrier function [18,19,20,21]. Hence, we investigated the changes in the PPARγ, MAPK, and NF-ĸB signaling pathways following CUM treatment.

**Figure 5:**
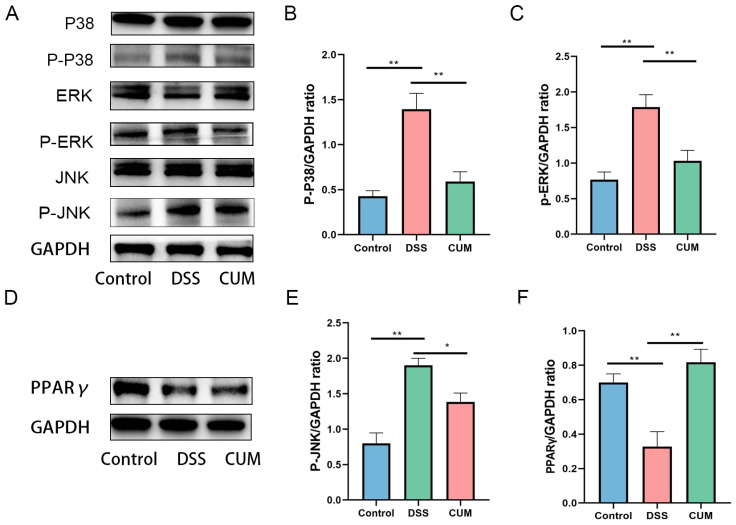
CUM protects against colitis via MAPKs inhibition and PPARγ activation. Quantitative results show that CUM inhibits the activation of (**A**) p38 (**B**), P-ERK (**C**) and P-JNK (**E**) in DSS-treated mice. (**D**) Representative images show that CUM promotes the nuclear translocation of PPARγ. (**F**) Relative protein expression ratios of PPARγ. Each value is expressed as mean ± SD (n = 6). Significance = * *p* < 0.05 and ** *p* < 0.01 indicate a significant difference compared with the DSS group.

Based on the Western blotting results shown in Figure 4D–F, the phosphorylation levels of NF-κB p65 and IκBα were upregulated after DSS intake, while CUM significantly downregulated the phosphorylation levels of NF-κB p65 and p-IκBα (*p* < 0.05).indicating that activation of the NF-κB signaling pathway is inhibited by CUM. Additionally, following CUM intervention, the expression of proteins mediated by the MAPK signaling pathway was also significantly downregulated. As expected, following DSS treatment, the relative expression levels of p-p38, p-ERK, and p-JNK significantly increased, while CUM reduced the expression of these proteins (Figure 5A–E). Additionally, we observed PPARγ activation in UC mice treated with CUM, manifested by an increase in the proportion of PPARγ located in the nucleus (Figure 5F). Taken together, these findings indicate that CUM activates PPARγ and inhibits the activation of the MAPK and NF-κB signaling pathways.

### 2.6. CUM Altered the Intestinal Microbiota Composition in Colitis Mice

**Figure 6:**
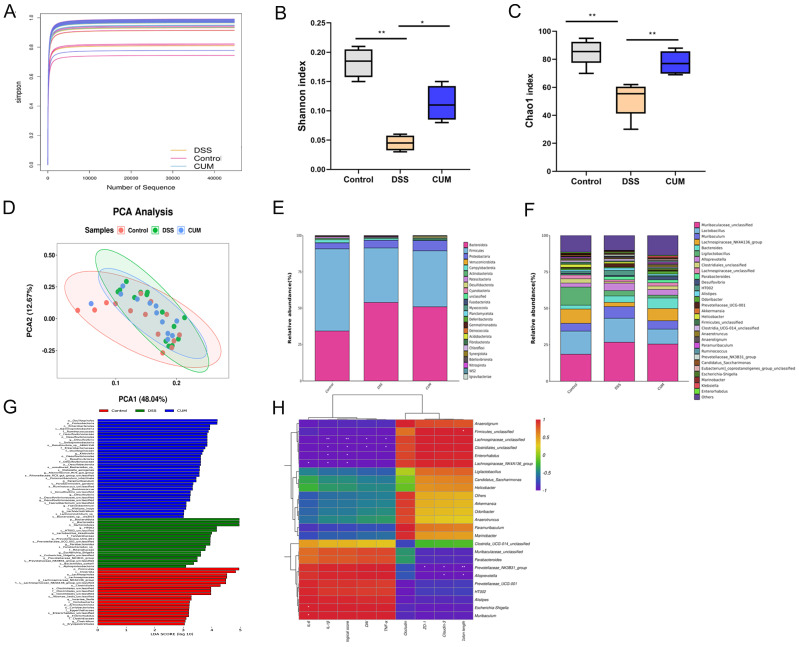
CUM Supplementation Effects on Gut Microbiota Diversity. (**A**) Simpson curve; (**B**) Shannon index; (**C**) Chao1 index; (**D**) PCoA plot. Changes in the colon gut microbial community following DSS administration (**E**) at the phylum level and (**F**) at the genus level. (**G**) Distribution histogram of gut dominant microorganisms by genus. (**H**) Correlation heatmap analysis using the Spearman correlation coefficient to determine the relationship between intestinal bacteria and the severity of colon length, DAI score, histopathology score, IL-β, IL-6, TNF-α, Occludin, and Cluadin-3. Data are presented as mean ± SD (n = 6). Significance = * *p* < 0.05 and ** *p* < 0.01 indicate a significant difference compared with the DSS group.

All groups’ sparse curves tended to stabilize, indicating sufficient sequencing coverage for the samples (Figure 6A). A significant reduction in α-diversity was observed (*p* < 0.05) in the DSS-treated group compared to the Control group. However, after CUM treatment, there was a notable recovery in the richness of the gut microbiota in the mice, exhibiting a significant increase compared to the DSS-treated group (Figure 6B,C). Changes in the β-diversity of the gut microbiota were further observed through PCOA analysis (Figure 6D). There was partial overlap between the DSS group and the CUM group in the PCoA plot, indicating a high degree of similarity in the overall structure of the gut microbial community between the two groups. This suggests that CUM may exert its intervention effect by regulating key species rather than the overall community structure.

At the phylum level, in the intestinal microbiota of mice, *Firmicutes* had the highest relative abundance, followed by *Bacteroidota*, with a relative abundance of 51.92% and 39.10% (Figure 6E). Compared to the control group, the relative abundances of *Firmicutes*, *Proteobacteria*, and *Campylobacterota* in the DSS group increased by 6.72%, 2.60%, and 2.00%, respectively. At the genus level, the relative abundance of *Lactobacillus*, *Bacteroides*, and *Ligilactobacillus* in the DSS group decreased by 0.79%, 2.28%, and 2.84%, respectively (Figure 6F). Following the gavage intervention with *CUM*, there was a significant increase in the relative abundance of these genera in the colonic contents of mice. Conversely, the relative abundance of *Anaerotruncus* increased by 3.04% in the DSS group compared to the control group. The LEfSe analysis corroborated these findings, indicating that supplementation with CUM partially restored most of the influential microbiota to levels observed in the control group (Figure 6G). Correlation analysis showed a significant positive correlation between *Escherichia-Shigella* and IL-6 levels in the colon (*p* < 0.05) (Figure 6H).

### 2.7. CUM Restored the Production of SCFAs in the Colon

**Figure 7:**
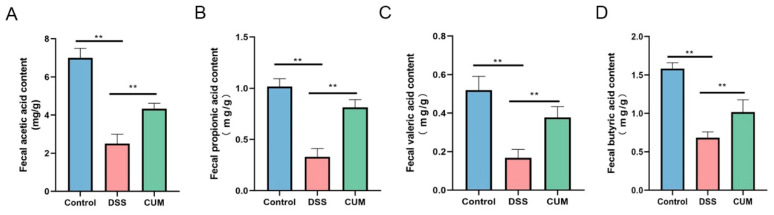
Analysis of SCFAs in feces. (**A**) The content of acetic acid in feces; (**B**) the content of propionic acid in feces; (**C**) the content of valeric acid in feces; (**D**) the content of butyric acid in feces. Data are presented as mean ± SEM (n = 6). Significance = ** *p* < 0.01 and indicate a significant difference compared with the DSS group.

According to prior reports, the progression of UC is associated with changes in the levels of SCFAs in the intestines. The levels of SCFAs in the feces and serum of UC patients are generally reduced, which is directly related to the decreased abundance of SCFA-producing bacteria such as *Faecalibacterium prausnitzii*. A deficiency of SCFAs can exacerbate intestinal inflammation and barrier damage, forming a vicious cycle. In our previous experiments, CUM was found to alter the relative abundance of SCFA-producing bacteria in UC mice. Therefore, the levels of acetic acid, propionic acid, butyric acid, and valeric acid in the mice were measured. As shown in Figure 7, compared with the control group, the concentrations of acetic acid, propionic acid, valeric acid, and butyric acid in the mice of the DSS group were significantly decreased (*p* < 0.05). However, after treatment with CUM, the levels of acetic acid, propionic acid, valeric acid, and butyric acid in the mice of the CUM group increased significantly (*p* < 0.05).

## 3. Discussion

UC is a chronic, nonspecific inflammatory bowel disease of unknown etiology. In addition to chronic nonspecific changes in the intestines, UC can lead to extraintestinal complications affecting multiple organs such as the liver, skin, and joints, with an incidence rate of 21% to 47% [19]. Currently, the main clinical treatments include aminosalicylates, glucocorticoids, and immunosuppressants. Although these medications can alleviate symptoms in the initial stages of treatment, relapses are common after discontinuation, often accompanied by significant adverse reactions, yielding suboptimal overall therapeutic effects. Aminosalicylates can only alleviate symptoms in 50% of patients with mild-to-moderate active UC [20]. Glucocorticoids can lead to severe side effects such as hypertension, glaucoma, and osteoporosis. Immunosuppressants such as thiopurine drugs and calcineurin inhibitors can cause liver and kidney damage [21]. It has been discovered that the metabolites of intestinal microbiota can modulate the host’s inflammatory response, providing a new approach and method for UC treatment [22].

Probiotics are living microorganisms that, when ingested in appropriate amounts, have beneficial effects on host health. To date, many probiotic strains have been studied in clinical trials for IBD [23]. In clinical trials of patients with inflammatory bowel disease (IBD), it has been found that supplementation with *Bifidobacterium* can reduce inflammatory markers and improve the clinical symptoms of patients, such as alleviating diarrhea and abdominal pain [24,25]. In recent years, studies have indicated the efficacy of various probiotics in the treatment of UC with relatively minor side effects, thus garnering attention for the value of probiotics in UC treatment [25]. The Food and Drug Administration (FDA) has approved the use of CUM as a safe organism for a wide range of applications, including the production of various bioproducts such as glutathione, specific amino acids, and enzymes [26]. Studies have shown that CUM promotes the development of intestinal immune organs, humoral and cellular immunity, and rapid clearance of endogenous pathogens, as well as improving the efficiency of the immune system, and enhances the resistance of animals to various diseases. As a probiotic, CUM can produce bacteriocins to inhibit the proliferation of harmful microorganisms such as *Escherichia coli*; it can also produce enzymes to prevent pathogenic bacteria from adhering to epithelial tissues [27]. Moreover, CUM can produce hydrogen peroxide and other nutrients to prevent the growth of Gram-negative bacteria during fermentation and to inhibit the accumulation of toxic substances. In this study, it was further demonstrated that CUM, by inhibiting weight loss, alleviating the reduction in colon length, and protecting against intestinal mucosal damage in mice with DSS-induced experimental colitis, might have the potential to treat IBD. Therefore, in this experiment, a UC model was constructed using DSS-induced mice, and the beneficial effects of CUM on the intestinal barrier and intestinal microbiota of UC mice were further investigated.

Intestinal barrier dysfunction primarily refers to an abnormal increase in permeability, allowing pathogens to cross the barrier, which is regulated by TJs formed between intestinal epithelial cells in the apical region [23]. Functional TJs are essential for maintaining intestinal permeability and intestinal barrier function [8]. The transmembrane proteins Occludin, Claudins, and ZO-1 are reported to be critical for regulating intestinal permeability. Intestinal barrier damage is considered to play a key role in the pathogenesis of UC [20]. Intestinal barrier damage leads to increased permeability, which subsequently facilitates the translocation of pathogens into the underlying vascular circulation [24]. Intestinal epithelial injury is considered to be the initiating event in chemically induced colitis models [25,26,27]. The results of this study indicated that DSS significantly reduced the expression of ZO-1, Claudin-3, and Occludin, while CUM increased their expression, thereby mitigating DSS-induced epithelial injury. Furthermore, our experimental results showed that DSS induced elevated serum levels of IL-6, IL-1β, and TNF-α, whereas these levels were reduced after CUM treatment.

KEGG enrichment analysis showed that the NFκB pathway plays a crucial role in the treatment of colitis in mice with CUM. The confirmation of reduced phosphorylation levels of NFκB p65 substantiates the inhibition of the NFκB pathway by CUM, indicating its role in controlling colitis. Additionally, expression validation through the MAPK and PPARγ signaling pathways suggests that CUM may also inhibit the MAPK and PPARγ signaling pathways to alleviate colitis in mice.

Previous studies suggest that the primary role of probiotics is to normalize the altered intestinal microbiota [28,29,30,31]. In this study, ecological dysbiosis of the intestinal microbiota following DSS treatment was determined using alpha and beta diversity compared to the control group. Abundant intestinal bacteria bind to intestinal epithelial cells, forming a robust barrier with the ability to resist the invasion of pathogens in the intestine [32,33]. In our experiments, differential abundant taxa were found among the Control, DSS, and CUM groups, including Firmicutes, *Lactobacillales* and *Roseburia* in the Control group and *Candidatus_Arthromitus, Clostridiales*, *Colidextribacter* and *Oscillibacter* in the DSS-treated mice. CUM treatment significantly restored the dysbiotic taxa induced by DSS. Interactions between the host and microbiota are primarily mediated by metabolites, which originate from bacterial metabolism of dietary substrates, modification of host molecules, or directly from bacteria, such as SCFAs [34]. In IBD patients, the levels of SCFAs and SCFA-producing intestinal bacteria are significantly reduced [35]. Our study found that compared to the control group, the abundance of SCFA-producing bacteria in fecal samples from the DSS group decreased, which was correlated with decreased SCFA levels, such as *Muribaculaceae*, *Bifidobacterium*, and *Lactobacillus* [32]. The changes in the abundance of SCFA-producing bacteria were likely to be responsible for the decreased levels of several major SCFAs, including acetic acid, propionic acid, and butyric acid. Additionally, SCFAs exerted immune-regulatory effects by increasing the number of intestinal Treg cells that maintain epithelial homeostasis. CUM was found to increase the abundance of SCFA-producing bacteria and fecal SCFA levels.

## 4. Methods and Materials

### 4.1. Chemicals

Dextran Sulfate Sodium Salt DSS (Cat#9011-18-1) was purchased from MP Biomedicals (Irvine, CA, USA). Specific antibodies, including anti-ZO-1 (1:1000; #AF5145; RRID:AB_2837631), anti-Occludin (1:1000; #DF7504; RRID:AB_2841004), anti-Claudin-3 (1:1000; #AF0129; #RRID:AB_2833313), anti-NF-κB p65 (1:1000; #BF8005:AB_2846809), anti-p-p65 (1:1000; AB_2834435:AF2006), anti-NF-κB IKBα (1:1000; #AF5002:AB_2834792), anti-NF-κB p-IKBα (1:1000; #AB_2834433:AF2002), anti-MAPK ERK (1:1000; #AB_2833336:AF0155), anti-MAPK p-ERK (1:1000; #AB_2834432:AF1015), anti-MAPK JNK (1:1000; #AF6318:AB_2835177), anti-MAPK p-JNK (1:1000; #AF3318:AB_2834737), anti-MAPK p38 (1:1000; #BF8015:Q16539), anti-MAPK p-p38 (1:1000; #AF4001:AB_2835330), anti-PPAR gamma (1:1000; #AF6284:AB_2835135) and anti-GAPDH (1:1000; #AF7021; AB_2839421) antibodies, were purchased from Affinity Biosciences (Cincinnati, OH, USA). TNF-α (Cat# 430915), IL-1β (Cat# 432615), and IL-6 (Cat# 431307) ELISA kits were obtained from Biolegend (San Diego, CA, USA).

### 4.2. Strain Preparation

*Candida utilis* (NO. CICC1769) was purchased from the China Center of Industrial Culture Collection (CICC, Beijing, China). The CUM strain was cultured in malt extract agar medium. The culture was then incubated overnight in a shaking incubator at 37 °C and 180 rpm. Upon completion of the incubation period, the bacterial culture was harvested by centrifugation at 3000 rpm/min for 15 min. This centrifugation step facilitated the separation of bacterial cells from the medium. Subsequent washing with PBS helped to remove any residual medium or impurities. Following the washing procedure, the bacteria were resuspended in phosphate buffered saline (PBS, pH = 7.4) to achieve a concentration of 1.0 × 10^9^ colony-forming units (CFU/mL) per milliliter, ready for further experimentation or application.

### 4.3. Animals and Experimental Design

This study was approved by the Animal Protection and Use Committee of Northwest Minzu University, Lanzhou, China (No: XBMU-SM-2020010). We used 27 male SPF C57BL/6J mice, aged 8 weeks and weighing 19–21 g, which were obtained from the Animal Experimental Center of Northwest Minzu University. After a 7-day adaptation period, the 27 male BALB/c mice were randomly divided into 3 groups, with 9 mice in each group: a control group (Control), an inflammation group (DSS), and a *Candida utilis* intervention group (CUM). The mice were housed at a temperature of 23 ± 1 °C and humidity of 50 ± 10%, with a natural light/dark cycle and ad libitum access to food and water. From Day 1 to Day 7, the control group was given water ad libitum, while the DSS and CUM groups were given a 2.5% DSS water solution. After freely consuming DSS for 7 days, the mice in the CUM group were orally administered CUM at a dose of 1.0 × 10^10^ CFU/kg/day for 7 days, while those in the DSS and control groups were orally administered saline for 7 days. At the end of the experiment, the mice were euthanized, and blood, colon, and fecal samples were collected on Day 8 and stored at −80 °C. This experiment was designed to investigate the effects of CUM on a mouse model of DSS-induced UC and to explore its potential therapeutic effects and mechanisms of action.

### 4.4. Disease Activity Index (DAI)

During the experiment, the following parameters were recorded daily for each mouse: body weight, fecal characteristics, and fecal occult blood status. To quantitatively analyze the recorded information, the DAI scoring method, as mentioned in the literature, was employed. The DAI scoring method typically involves the assessment of parameters such as weight loss, stool consistency, and the presence of fecal occult blood. Each parameter is assigned a score, and the sum of these scores yields the DAI for each mouse. This method allows for quantitative assessment of disease severity and progression in the experimental mice. The DAI score, ranging from 0 to 12, was calculated as the sum of scores from three parameters. The DAI was determined according to the average scores of weight variation, stool consistency, and bleeding amount, following the scoring system described in a previous study [35]. Each measurement was replicated six times.

### 4.5. Histological Analysis of Tissues

The colon tissue samples were fixed in 4% paraformaldehyde and routinely processed to prepare paraffin-embedded tissue sections (5 μm). Following H&E staining, histopathological examination was conducted using an optical microscope (Nikon Eclipse E100, Tokyo, Japan). Two pathologists carried out histological scoring in a double-blind manner. The severity of colitis was evaluated according to the cumulative scores of three aspects: the infiltration degree of inflammatory cells (scored from 0 to 4), the presence and degree of ulceration (scored from 0 to 4), and the extent of crypt distortion area (scored from 0 to 4).

### 4.6. Measurement of Oxidative Stress Level and Inflammatory Cytokine Levels

Colon tissue was collected from each group of mice, and a 10% homogenate was prepared with PBS. According to the manufacturer’s instructions (Jiancheng, Nanjing, China), the colon of each group of mice was detected using an assay kit (Jiancheng, Nanjing, China). The serum levels of cytokines IL-1β, IL-6, IL-8, and TNF-αwere determined according to the ELISA kit instructions (San Diego, California, USA). The absorbance was measured at 450 nm.

### 4.7. Western Blotting

According to the current protocol, the colon tissues were homogenized in RIPA lysis buffer. Subsequently, the samples were normalized to the same protein concentration using a BCA assay kit (Solarbio, Beijing, China). Protein samples (30 µg) were separated by electrophoresis on a 15% SDS–polyacrylamide gel and then transferred onto a PVDF membrane. After blocking with 5% skim milk, the membrane was incubated overnight at 4 °C with the primary antibodies, followed by a 60 min incubation with horseradish peroxidase (HRP)-conjugated secondary antibodies. The expression of ZO-1, Occludin, Claudin-3, p38, p-p38, JNK, p-JNK, ERK, p-ERK, NF-κB p65, p-p65, IκBα, p-IκBβ, and GADPH was detected. The protein blot bands were quantitatively analyzed using ImageJ software 6.0 [11].

### 4.8. Immunofluorescence Staining

The colon samples were fixed in 4.0% formaldehyde and then sectioned into 5 μm slices. These slices were permeabilized with 0.25% Triton X-100, followed by tissue blocking with 5.0% FBS, and then incubated with primary antibodies against ZO-1. After washing, the slices were incubated with fluorescently labeled secondary antibodies. Subsequently, all images were observed and captured using an inverted fluorescence microscope.

### 4.9. 16S rRNA Analysis

To analyze the microbiota in the colon content, colon content samples (100 mg each) were taken. Total DNA was extracted from the intestinal contents using the E.Z.N.A. Stool DNA Kit (*Omega Bio-tek, Norcross, GA, USA*). The quality of DNA extraction was assessed by agarose gel electrophoresis, and DNA was quantified using a UV spectrophotometer (Thermo Fisher Scientific, Waltham, MA, USA). The PCR products were purified using AMPure XT beads (Beckman Coulter, Brea, CA, USA) and quantified using Qubit (Thermo Fisher Scientific, Massachusetts, USA). Sequencing was performed using an amplicon pool (Agilent Technologies, Santa Clara, CA, USA), and the size and quantity of the amplicon library were measured using an Agilent 2100 Bioanalyzer (Agilent Technologies, California, USA.) and Illumina’s library. The library was sequenced using the NovaSeq PE250 platform (Illumina, San Diego, CA, USA).

### 4.10. RNA Sequencing

RNA extraction, cDNA library construction, and RNA sequencing (RNA-seq) were carried out. Total RNA was extracted from mouse colon samples using TRIzol (*Solarbio, Beijing, China*), with three biological replicates. Commercial library preparation and sequencing were performed by Majorbio Biotech (Shanghai, China) on an Illumina HiSeq 2000. The raw sequencing data were processed by trimming the adapters and removing low-quality reads. Short sequence reads were analyzed on the Majorbio I-Sanger Cloud Platform. Differentially expressed genes (with a fold change ≥2 and FDR < 0.05) associated with biological processes, cellular components, and molecular functions were analyzed based on GO annotation. Immune response-related genes were analyzed based on the KEGG pathway. GO and KEGG enrichment analyses of DEGs were performed using *p* < 0.01 as the significant enrichment level, and the expression levels of DEGs were determined using FPKM values.

### 4.11. Determination of Short-Chain Fatty Acids (SCFAs)

The SCFAs in fecal samples, including acetate, propionate, butyrate and valerate, were quantitatively determined by ion chromatography. Briefly, 50 mg of fecal sample was added to 100 μL of 15% formaldehyde, 100 μL of formaldehyde (125 μg/mL internal standard solution) and 900 μL of ethylene glycol. The supernatant was diluted (1:50). Then, it was centrifuged at 3000× *g* for 10 min. The supernatant was taken, filtered through a 0.22 μm membrane for sterilization, and stored in a 2 mL screw-cap vial. Subsequently, the SCFAs were analyzed using an ion chromatography system (Thermo Fisher Scientific, Waltham, MA, USA).

### 4.12. Statistical Analysis

The experimental results were statistically analyzed by one-way analysis of variance (ANOVA) using SPSS 27.0 software. The results are presented as mean ± standard deviation (mean ± SD). Pearson correlation analysis was used to examine the correlations between the variables, with 95% confidence intervals. *p* < 0.05 was considered statistically significant.

## 5. Conclusions

CUM can alleviate DSS-induced colitis in mice by reducing inflammatory cytokines and oxidative stress. CUM can mitigate the progression of DSS-induced colitis in mice by inhibiting the activation of the NF-κB/MAPK signaling pathway. Additionally, CUM is hypothesized to partially regulate the intestinal microbiota by increasing the diversity of beneficial bacteria and reducing the relative abundance of pathogenic bacteria. The increased diversity of beneficial bacteria and their metabolites may modulate inflammatory cells, thereby protecting the intestines from DSS-induced inflammation. This study provides a theoretical reference for further research on the clinical application of CUM as a therapeutic agent for the prevention and treatment of gastrointestinal reactions associated with UC.

## Data Availability

All related data and methods are presented in this paper. Additional inquiries should be addressed to the corresponding author.
